# Comprehensive Analysis of a Tricycle Structure with a Steering System for Improvement of Driving Properties While Cornering

**DOI:** 10.3390/ma15248974

**Published:** 2022-12-15

**Authors:** Miroslav Blatnický, Ján Dižo, Denis Molnár, Andrej Suchánek

**Affiliations:** Department of Transport and Handling Machines, Faculty of Mechanical Engineering, University of Žilina, Univerzitná 8215/1, 010 26 Žilina, Slovakia

**Keywords:** three-wheeled vehicle, vehicle frame, stability, simulation, material fatigue, structural design

## Abstract

This paper focuses on the development, theoretical and experimental research on the structural units of an unconventional three-wheeled vehicle. The vehicle is designed in order to increase the stability when cornering in a low curvature radius. Current research work describes solutions to increase the cornering stability of either conventional three-wheeled vehicles or, more rarely, unconventional vehicles designed on the basis of complex wheel-tilting mechatronics. Thus, there is a gap in research in respect of consideration of a stability-enhancing mechanism for three-wheeled vehicles based on a combination of tilting and deflection of the front steered wheel in the course of cornering. This paper then compares the stability of a three-wheeled vehicle with one steered wheel in front and two wheels in the rear (1F2R) in conventional and unconventional designs. A particular linear formula for the stability of the three-wheeled vehicle in cornering is derived. This study further deals with the design of the frame intended to hold the unconventional steering mechanism of the front wheel of the vehicle, on the one hand, from the theoretical integrity point of view using CAD-, FEM- and MBS-based software and, on the other hand, from the experimental point of view by determining the multiaxial fatigue life of the test specimens. These were made from the frame structural material and loaded with an equivalent load (bending-torsion) corresponding to the real load of the frame in operation. It was discovered that the designed patented front wheel steering mechanism increased the passing speed by 19% in comparison with a conventional vehicle at the minimum possible radius of a corner. The designed vehicle meets the safety conditions in terms of frame integrity and load-bearing capacity. The vehicle frame is designed with respect to the fatigue life of the material, the results of which are presented in the work. The material employed for manufacturing the frame is aluminum alloy type EN AW6063, which makes the frame lightweight and strong.

## 1. Introduction

Three-wheeled vehicles are today a commonly used means of transportation in everyday life, practice, or sport. Their classification is regulated in accordance with the national ordinances of individual countries. Within the Slovak Republic, the regulations contain the conditions that three-wheeled vehicles must meet standards for authorized movement on the roads [1].

It is estimated that around 300 million people in the EU have a driving license. Drivers have to concentrate on the surrounding traffic at all times when driving. This is not only related to their safety, but also to the safety of passengers and other road users [2].

Generally, everyone can contribute to improving road safety, for example, by means of obeying traffic rules. Speeding is one of the most influential factors affecting road safety. It sharply increases the risk of a road accident. Moreover, it plays a significant role in approximately 30% of fatal accidents [3]. In terms of industrialized countries, traffic accidents are the most common cause of occupational fatalities. It is a well-known fact that if all drivers wore seat belts, did not exceed the speed limit, and did not drive under the influence of alcohol, more than 12,000 lives would be saved annually on European roads. Globally, road traffic injuries are presently the eighth leading cause of all deaths [4].

Three-wheeler rollover crashes account for more than one-third of the total number of fatalities and serious injuries, albeit they represent a small proportion of the total number of crashes [5]. A conducted comparison of two-wheeler and three-wheeler crashes with car crashes demonstrated that three-wheeler and two-wheeler crashes are more severe [6]. Therefore, contributing to improve (raise) road safety is one of the objectives of this paper. This can be done in several areas.

**Figure 1:**
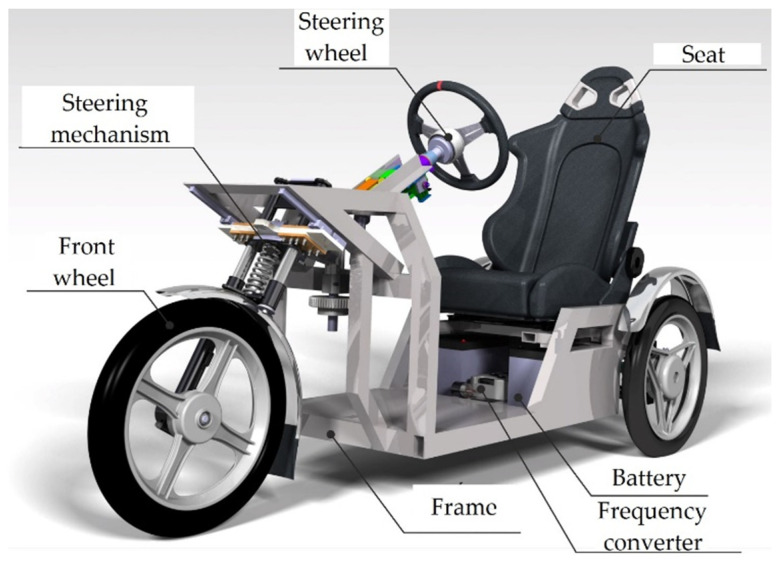
Three-dimensional CAD model of the prototype—E3-cycle.

The primary area is the design essence of the solved means of transport, the so-called “E3-cycle” (Figure 1). The static stability factor (SSF) is one of the chief parameters for comparing the rollover probability of vehicles. It is a ratio between half of a vehicle’s track width and the height of the center of gravity of the vehicle. In the case of a conventional three-wheeled vehicle (delta type), this ratio is smaller than in the case of a four-wheeled vehicle of similar size, by a factor equivalent to the ratio of the center of gravity distance to the rear axle [7]. Thus, for the same track width and center of gravity height, a three-wheeled configuration is more prone to rollover than a four-wheeled structure of comparable size [8]. In paper [9], it was found that the rollover propensity of a three-wheeled vehicle is almost double in contrast to the least stable four-wheeled SUV. On the basis of the limits of lateral acceleration, four-wheeled vehicles are evidently more susceptible to directional instability than rollover in the course of rollover (compared to three-wheeled vehicles).

In a paper [10], the authors put forward the design of a patented front-wheel steering system mechanism for this vehicle [11]. It is a completely new idea of a steering system of a front wheel of a three-wheeled vehicle. The main advantage of the system in comparison with existing technical solutions is the working principle itself. This steering system works completely on a mechanical principle only with a specific gear system. Other systems for improving driving safety in curves combine various supporting electronic and control systems. On the other hand, this system still has a disadvantage resulting from a change of the center of gravity. The system does not have a self-returning effect. It means the driver needs to generate an additional force (or torque) to return a three-wheeled vehicle to a straight direction.

The intention was an expected substantial increase (results were not published in the paper) in the stabilizing effect when the E3-cycle was cornering. This would allow cornering at higher speeds in contrast to conventional three-wheelers. However, the improvement in riding characteristics obtained in such a way should not only lead to fewer violations of traffic regulations. It is expected to be an element of active vehicle safety to help prevent accidents during which the rider is taking a corner at a potentially dangerous speed (hazardous for conventional three-wheelers). This area (of increasing the cornering stability of three-wheelers) is involved in the presented paper in the form of creating linear mathematical models of a conventional and an unconventional vehicle and comparing them.

The second area, namely environmental impact, was addressed in a paper [12], where the authors described the development of an E3-cycle frame that was made of a lightweight aluminum alloy. The use of this material resulted in a significant saving of mass (up to 40 kg in contrast to a steel frame) and a reduction in the position of the center of gravity of the vehicle by up to 150 mm. In addition, this has a positive effect on the environment on the one hand, and on the stability of the vehicle itself on the other. These days, an emphasis should certainly be placed on reducing carbon footprint. The E3-cycle project is an alternative to current means of transport, especially in urban transportation or on short routes with heavy traffic. In terms of contrast to conventional means of transport, it offers more solutions to the global warming issue due to its electric propulsion. This area is involved in the presented paper in the form of numerical simulations of a vehicle-supporting frame specially adapted for the patented unconventional front wheel steering mechanism of the E3-cycle. A.A. Dere et al. [13] used modal analysis to find the maximum deformation in the chassis solved by them. In a paper [14], they reported ways to improve the three-wheeled chassis by means of strain analysis. The research was focused on reducing the weight and manufacturing costs. Moreover, they pointed out the results achieved. The authors in a paper [15] designed a chassis for electric motorcycles with regard to emission standards and structural stability. Accordingly, the presented paper also sets out to analyze the frame design of the E3-cycle. The development of this structural unit was carried out using CAD software, namely Catia (Dassault Systèmes, Vélizy-Villacoublay, France). After importing the model into Ansys FEA analysis software (Ansys Inc., Canonsburg, PA, USA), a finite element analysis (FEA) was carried out.

The research went on to the third area and was published in a paper [16], where the authors were concerned with the multiaxial fatigue life of the vehicle frame material. It was ascertained that the particular motion capabilities of the steered wheel cause specific stresses on the frame and its welds. Ultimately, the current work aims to analyze the distribution and magnitudes of the loads on the vehicle frame in the course of its operation (determined by FEM simulation). Furthermore, it is intended to complement other experimental results from fatigue measurements that have not been as yet published. On this basis, it will be possible to determine whether the operation of the vehicle design is safe from the point of view of integrity, load capacity, and durability [17,18,19]. Thus, it will be feasible to design this structural unit with maximum profitability.

In the sense of theoretical level, models of a three-wheeled vehicle with standard control and models of the E3-cycle will be created by means of analytical and numerical calculations. The most important part of the first area will then be the evaluation of the obtained results for both types. By dint of mathematical models that will describe the dynamic properties of the individual vehicles compared, the maximum speeds at the time of the vehicle passing through corners with various radii of curvature will be determined. In this way, the effect of the increase in stability will be theoretically verified. In terms of a logical progression, the authors’ objective is to bring the proposed vehicle to a state of possible standardization, thus enabling both its serial production and commercial use.

The frame stress results obtained in the second area of the paper will serve as input for the experimental research of multiaxial (bending-torsion) fatigue life from the third area. The samples will be loaded with the same stresses as the vehicle frame is loaded with; in other words, with stresses obtained through simulations.

The outcome after the end of the research should be an implementation of scientific knowledge into a real product. Therefore, it is necessary to analyze the issue comprehensively together with the mechanics and the vehicle operator. For this reason, Section 2 has been included in the paper. It explains the substantial problems and the scientific approach to their solutions in conjunction with a practical view of the issue of this unconventional vehicle.

## 2. Problem Statement

**Figure 2:**
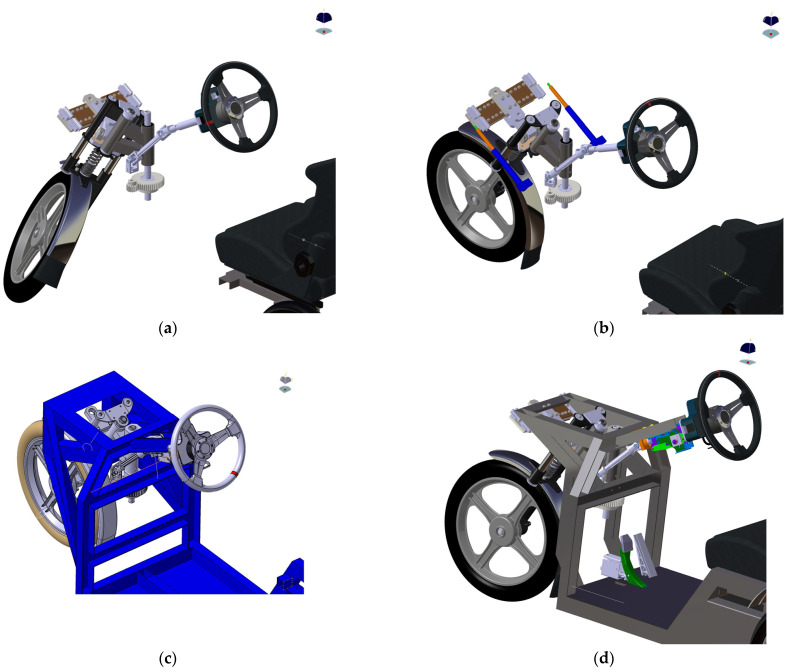
A 3D CAD model of the mechanism design: (**a**) Original design; (**b**) Design no. 1; (**c**) Design no. 2; (**d**) Design no. 3.

In a paper [16], it was reported that after the building of a real prototype, an improper behavior of the vehicle was observed due to the creation of the singularity in the central position (when the vehicle is riding straight) resulting from the nature of the mechanism (Figure 2a). It is important to highlight that the authors are currently dealing with a study that is specifically tasked with the MBS simulations of E3-cycle riding modes. The SIMPACK simulation tool used is partially limited in its ability to address this phenomenon. As a consequence, from a pragmatic point of view of the problem, a design modification [16] (Figure 2b) was proposed to keep the conditions of low production costs and minimal intervention in the frame design. In the event that this modification would not be sufficient (it has not yet been practically implemented in a prototype), two further optimizations of this condition were proposed.

Thus, in overall order, the second design (Figure 2c) was made up of a structural change to the frame. The front part of the frame and its attachment to the rest of the structure were changed. The initial idea behind this change was an attempt to rotate the linear bearings by a vertex angle about the zero position (the determined zero position is parallel to the ground). The purpose of this design was that there would be a small lift of the front of the frame when turning. Accordingly, gravity would ensure that the front wheel would return to a straight line. The mechanism of action of the force is similar to that of Design 1, as the steering system has not been significantly intervened. The advantage of this type of design is the increased stability when cornering and the ability to achieve a straight line after releasing the steering wheel. The major disadvantage is the complexity of the design and the concomitant need to intervene in the existing frame structure.

The considerable design modification is represented by Design 3 (Figure 2d), where the steering attachment has been altered. The steering tilting arm, linear bearings, and guide bar for linear bearings have been removed from the original design. In addition, the large gear wheel and pinion mounting were modified. The pinion was moved to the axis of the steering column. The large gear was redesigned as a “gear half-wheel”. Additionally, new brackets were designed to mount the pinion and the front wheel steering shaft. As a result of the removal of the steering tilting arm, the front fork axis of rotation was moved towards the rider. The steering mechanism of this design is based on the fact that the axis of rotation of the front wheel has been shifted to the axis of rotation of the steering shaft. Inasmuch as the degrees of freedom of the front fork rotation axis were removed, only rotation of the front fork occurred instead of the original semicircular motion. The steering wheel mounting in this design is the same as in Design 1 and Design 2. Thus, it is attached to the frame with a steering column bracket. Similarly, the steering shaft is mounted in ball bearings. The steering column and steering shaft are connected by means of a universal joint. However, the modification is that the steering shaft is mounted in a newly designed bracket that is tilted 33° against the pad compared to the first two designs. The bracket lies in the axis of symmetry, which in practical terms means that only one universal joint is necessary. The pinion is firmly attached behind the bracket via a steering shaft. The pinion transmits the force to the ‘gear half-wheel’ by means of gear transmission. The ‘gear half-wheel’ is fastened via a fixing pad to the new rotating shaft, which lies in the axis of symmetry. Furthermore, the shaft is attached to the fork using head bearings and concurrently to the frame structure using the new bracket. The bracket can be attached to the frame by either a bolted or welded joint. The fork does not perform a semicircular movement due to the design changes described above. This improves the riding characteristics with regard to riding in a straight line and stability when riding in a straight line.

All of the introduced design modifications represent independent patent solutions that improve the riding properties of conventional three-wheelers. However, their common indicator is the structural intervention (whether to a greater or lesser extent) in the original structural unit. Nonetheless, the authors strive to be able to eliminate the adverse effect of the singularity of the mechanism without affecting the original structure. Therefore, a concept has been designed that retains excellent price competitiveness.

**Figure 3:**
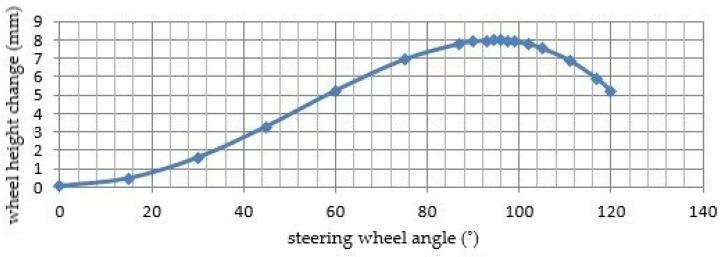
Graph of dependence of front wheel height on steering wheel angle.

A graph (Figure 3) representing the dependence of the vertical position of the front steered wheel on the steering wheel rotation was constructed from the 3D CAD model of the vehicle. It can be clearly seen from this dependence that in straight riding the wheel is at zero height (in such a way the origin of the coordinate system is chosen). Inasmuch as the wheel performs a shift and tilt in addition to the rotation, a difference in the height of the front part occurs (and hence the position of the center of gravity of the vehicle); i.e., it decreases.

Figure 3 demonstrates that the height of the front wheel of the E3-cycle decreases until it reaches a maximum deflection of 8 mm at *ζ* = 96° of the steering wheel rotation. Larger angles result in a slight decrease in deflection.

**Figure 4:**
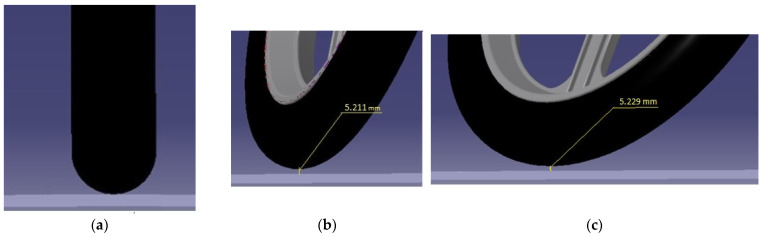
A change of wheel height when turning the steering wheel of the E3-cycle: (**a**) Driving in a straight track; (**b**) A partial front wheel deflection; (**c**) A full front wheel deflection.

**Figure 5:**
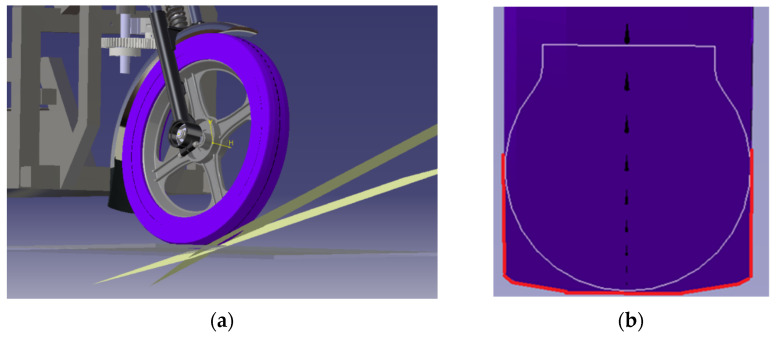
Forming of tire profile: (**a**) Formation of the geometry of the casing of the new tire; (**b**) Difference in the shape of the tire casing after the designed modification.

On the basis of the ascending curve (Figure 3) and visualization (Figure 4), the shape of the tire casing of the wheel was modified by simulation in Catia V5-6R2019 (Figure 5a). A result of this step was a suitable tire shape (Figure 5b) that ensures a constant height (zero change) of the vehicle’s center of gravity position. The principle of simulation consisted in placing the wheel on the plane of the pad in a straight direction. Subsequently, the wheel was turned in accordance with the motion capabilities of the steering mechanism. The contact (tangential) planes (Figure 5a) of each steering wheel rotation angle formed a new tire profile (Figure 5b).

**Figure 6:**
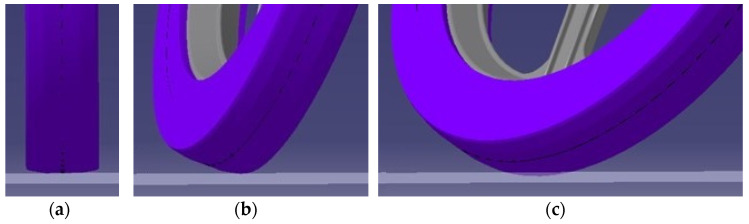
Position of the modified tire casing relative to the reference height when the steering wheel is turned at: (**a**) 0°; (**b**) 60°; (**c**) 90°.

Accordingly, the shape of the tire is adapted to the original wheel rim and the fork. Moreover, it is characterized by the self-centering effect of the vehicle during straight-ahead running due to the fact that it makes contact with the road at the same height as the center of gravity (Figure 6). The manufacturability of the tire is attainable, as assessed in consultation with the company Continental Matador Rubber, Ltd., (Púchov, Slovakia).

The new tire casing results in a 1.3-fold gain in moment of inertia compared to the original wheel (as determined through Catia software). The wider tire tends to raise resistance when turning the steering wheel. Generally, this causes faster tire wear. For that reason, an MBS simulation was performed using Simpack software, resulting in knowledge of the wheel force for a conventional three-wheeler and an E3-cycle of the same geometry when riding through a corner with a radius *R_z_* = 30 m.

**Figure 7:**
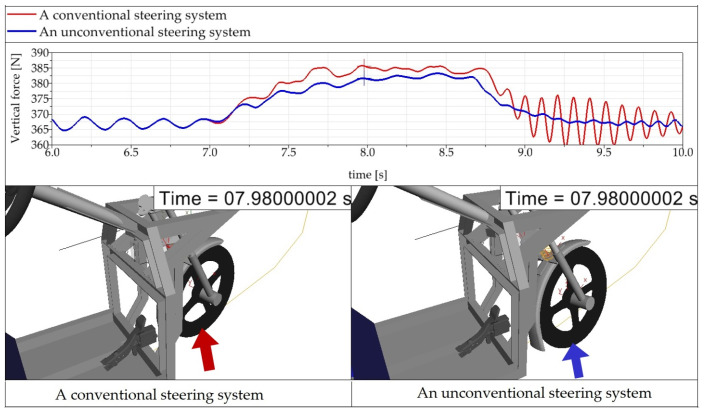
Total vertical wheel force of the front steered wheel at the time of riding through a left-hand bend by a three-wheeler with a conventional steering system and with an unconventional steering system.

It is evident from Figure 7 that in spite of the different steering mechanism of the front wheel, the total wheel force changes only slightly in service. In the case of riding the E3-cycle on a left-hand bend, the wheel force curve is smoother in contrast to the conventional three-wheeler (Figure 7).

It can be seen from the graphs (Figure 7) that behind the corner, the oscillation of the front wheel is smaller for the E3-cycle (time 8.75 s to 10 s), which has a more favorable effect on the safety of the ride along with the ride comfort. Similarly, the smoother front wheel force curve on the corner (time 7.0 s to 8.75 s) is conducive to greater ride safety and ride comfort for the E3-cycle compared to the conventional three-wheeler.

**Figure 8:**
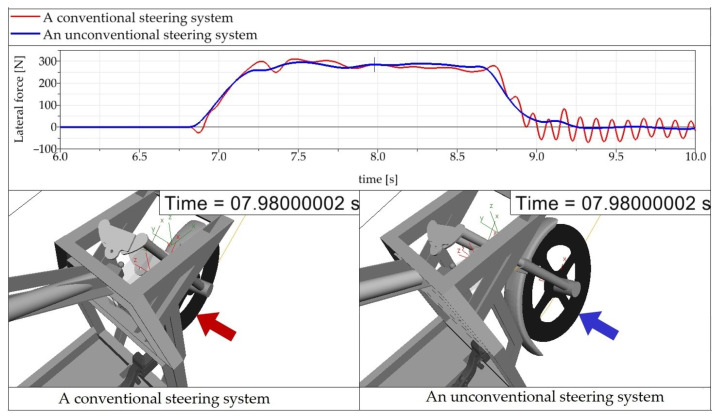
Total lateral wheel force of the front steered wheel at the time of riding through a left-hand bend by a three-wheeler with a conventional steering system and with an unconventional steering system.

Figure 8 shows a comparison of waveforms of the lateral wheel forces of the three-wheeled vehicle with a conventional steering system and with an unconventional steering system. In this case, it is also detected that the waveform of the lateral wheel force of the unconventional steering system is smoother (a blue curve) in comparison with the conventional steering system (a red curve). Moreover, the waveforms reveal that driving the vehicle with the unconventional steering system is safer, because driving the vehicle with the conventional steering system involves a more significant change in the lateral force amplitude (the time interval 8.75 s to 10 s, Figure 8). The investigation of the driving stability of the vehicle would exceed the range as well as the purpose of this paper. More detailed information is described in Section 5.

With respect to the presented vehicle loads, the lifetime of the unconventional tire casing of the front steered wheel designed by the authors is not expected to be significantly reduced. All the aforementioned modifications have a fundamental impact on the finalization of the research project as well as the implementation of the prototype for commercial use.

## 3. Materials and Methods

The quality of a vehicle depends primarily on its level of safety and reliable handling. The vehicle must respond sufficiently and within a short time interval to the operator’s suggestions. Otherwise, the vehicle does not meet the conditions to be brought into road traffic [20].

### 3.1. Mathematical Model of the Stability of a Conventional Vehicle and the E3-Cycle

**Figure 9:**
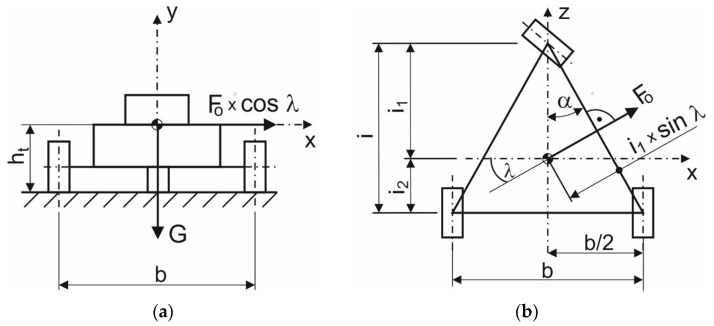
A calculation schema of a conventional tricycle: (**a**) Back view; (**b**) Top view.

The basic control of the steering mechanism in an electric tricycle with conventional steering is the axial rotation of the wheel as well as in motorcycles or bicycles. Single-track vehicles, in the course of cornering, compensate for the effects of centrifugal force by tilting in the opposite direction of that force. Inasmuch as the rear axle of the E3-cycle is two-wheeled, tilting is almost completely limited. As a result of noncompliance with the conditions, namely, the speed appertaining to a given turning radius, the centrifugal force may reach a critical value, which the vehicle-rider system will no longer be able to overcome. Consequently, the stability conditions will be violated. In terms of these undesirable circumstances, the vehicle is at risk of overturning. The necessary factors considered in the calculation include the wheel track, the weight distribution of the vehicle components or the approximate position of the total center of gravity, the rider ergonomics, and the design of the vehicle [21]. The mathematical notation of the above emerges from the schema depicted in Figure 8. The stability condition for a conventional three-wheeled vehicle is given by Formula (1):

(1) $$G\cdot i_{1}\cdot \sin\alpha \geq F_{o}\cdot \cos\lambda \cdot h_{t},$$

where *G* is the weight of the vehicle; *F_o_* is the centrifugal force during riding on a curve and *i*_1_, *α*, *λ*, and *h_t_* are the parameters of the vehicle depicted in Figure 9.

The congruence of angles *α* and *λ* emerges from Figure 9b:

(2) α=λ,

where *α* is the constant angle given by the design of a conventional three-wheeled vehicle; *λ* is the constant angle given by the design of a conventional three-wheeled vehicle.

Equation (3) applies for the vehicle’s center of gravity:

(3) G=m⋅g,

where *G* is the weight of the tricycle; *m* is the mass of the vehicle; *g* is the gravitational acceleration.

Equation (4) applies for the centrifugal force of the vehicle *F_o_*:

(4) $$F_{o}=m\cdot a,$$

where *F_o_* is the centrifugal force acting on the vehicle during cornering; *m* is the mass of the vehicle; *a* is the acceleration of the vehicle.

Equation (5) is applied for the acceleration of the vehicle *a*:

(5) $$a=\frac{v^{2}}{R_{z}},$$

where *v* is the instantaneous speed of the vehicle and *R_z_* is the radius of a corner.

Applying Equations (2)–(5) into Formula (1), we get (6):

(6) $$g\cdot i_{1}\cdot \sin\alpha \geq \frac{v^{2}}{R_{z}}\cdot \cos\lambda \cdot h_{t}.$$

Subsequently, the Formula (6) for calculating the maximum speed *v_max_* at which the vehicle can move on a bend with a given radius *R_z_* is obtained from the Formula (7):

(7) $$v_{max}\leq \sqrt{\frac{g\cdot R_{z}\cdot b\cdot i_{1}}{2\cdot h_{t}\cdot i}},$$

where *v_max_* is the maximum (safe) speed of the vehicle moving on the bend; *g* is the gravitational acceleration; *R_z_* is the radius of the corner; *b* is the track width of the vehicle; *i*_1_ is the distance of the vehicle’s center of gravity from the centerline of the front wheel; *h_t_* is the height of the vehicle’s center of gravity above the ground; *i* is the wheelbase of the vehicle.

**Figure 10:**
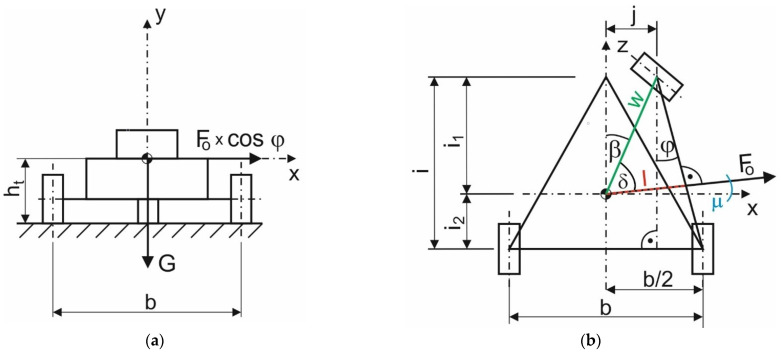
A calculation schema of the E3-cycle: (**a**) Back view; (**b**) Top view.

The steering mechanism of the E3-cycle differs from the conventional configuration in particular in terms of riding dynamics. By virtue of the action of the gearing, which connects the suspension of front wheel or more precisely steered wheel with a suitably designed structure, the steering axle is able to deviate relative to the vehicle’s central axis by a value of “*j*” (Figure 10), namely in the process of front wheel turning during the steering wheel turn. This compound motion increases the length of arm l through which the vehicle’s gravitational force is applied. As a result, the value of the moment of gravitational force *G* rises. This is a mechanism that affects the stability of the vehicle. The notation *j* represents the value by which the front wheel is extended to the outside of the curve. The value of *j* varies with the steering wheel angle *ζ*. Wheel extension occurs while the wheel is turning. The E3-cycle is able to make a passage on the curve with a minimum value of curve radius of *R_z_* = 1.5 m, whereby it is necessary to turn the steering wheel by *ζ* = 140°. In that case, the wheel deviates by *j* = 323.9 mm.

**Figure 11:**
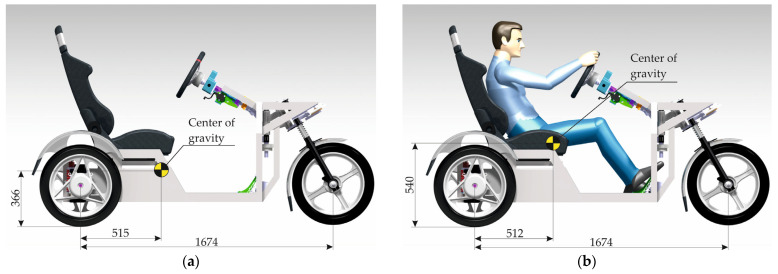
Position of the center of gravity: (**a**) The E3-cycle; (**b**) E3-cycle along with the model rider.

The center of gravity of the E3-cycle is located under the rider’s seat; therefore, closer to the rear axle of the vehicle, in the middle, as low as possible. The E3-cycle is fitted with wheels with a 260 mm radius tire. The position of the center of gravity is 93.9 mm above the wheel centerline (Figure 11). For the sake of summing these values, the position of the center of gravity from the pad is *h_t_* = 353.9 mm.

The way the E3-cycle passes through the curve affects its stability condition (8). By means of this mathematical scheme, it is possible to observe how large a centrifugal force effect the E3-cycle can overcome. The stability condition for the E3-cycle from Figure 10 is given by Formula (8):

(8) $$G\cdot l\geq F_{o}\cdot \cos\mu \cdot h_{t},$$

where *G* is the weight of the tricycle; *l* is the geometric variable dependent upon the parameter *w*; *F_o_* is the centrifugal force acting on the vehicle during cornering; *μ* is the variable angle dependent upon the value of the wheel deflection *j* (equivalent to the angle *φ*); *h_t_* is the height of the vehicle’s center of gravity above the ground.

Equation (9) emerges from Figure 10:

(9) l=w⋅cosδ,

where *w* is the variable dependent upon the value of the wheel deflection *j*; *δ* is the variable angle dependent upon the value of the wheel deflection *j*.

Moreover, the distance *w* is calculated from the Pythagorean theorem as (10):

(10) $$w=\sqrt{i_{1}^{2}+j^{2}},$$

where *i*_1_ is the distance of the vehicle’s center of gravity from the centerline of the front wheel; *j* is the variable distance of the deflection of the front wheel of the vehicle from the longitudinal axis of symmetry.

Further, the angle *δ* is calculated as follows:

(11) δ=90°−β−μ,

and the angle *β* is calculated according to Equation (12):

(12) $$\beta =arctg\frac{j}{i_{1}},$$

Finally, the angle *μ* is obtained using Equation (13):

(13) $$\mu =arctg\frac{\frac{b}{2}-j}{i},$$

where *μ* is the variable angle dependent upon the value of the wheel deflection *j* (equivalent to the angle *φ*); *b* is the track width of the vehicle; *j* is the variable distance of the deflection of the front wheel of the vehicle from the longitudinal axis of symmetry; *i* is the wheelbase of the vehicle.

Substituting Equations (9)–(12) into Formula (8), the Equation (14) is acquired:

(14) $$G\cdot l=m\cdot g\cdot \sqrt{i_{1}^{2}+j^{2}}\cdot \cos\left(90{}^{\circ}-arctg\frac{j}{i_{1}}-arctg\frac{\frac{b}{2}-j}{i}\right),$$

from where, after substituting Formulas (3)–(5), the Equation (15) for calculating the maximum speed *v_max_* is attained:

(15) $$v_{max}=\sqrt{\frac{g\cdot l\cdot R_{z}}{\cos\mu \cdot h_{t}}},$$

where *v_max_* is the maximum (safe) speed of the vehicle moving on the bend; *g* is the gravitational acceleration; *R_z_* is the radius of the corner; *l* is the geometric variable dependent upon the parameter *w*; *μ* is the variable angle dependent upon the value of the wheel deflection *j* (equivalent to the angle *φ*); *h_t_* is the height of the vehicle’s center of gravity above the ground.

Equations (7) and (15) are simplified analytical mathematical models. On the basis of these models, the analysis of the maximum speeds of the conventional and unconventional tricycle will be executed along with comparison of these speeds.

### 3.2. Numerical Simulation of Eigenfrequencies and Stresses of the E3-Cycle Frame

The frame of the E3-cycle is its basic supporting part. It has been designed to ensure the precise positioning of the individual structural units in order to prevent collisions and damage to the elements (reliable operation). The frame carries all types of loads [22]. Accordingly, significant requirements are placed on the E3-cycle frame based on safety and purposefulness. The shape, dimensions, and material are taken into account in addition to the strength properties and deformations of the frame. Consistency, integrity, eigenfrequencies, and eigenmodes were scrutinized in the graphical environment of Ansys 2019R3. The Ansys software is one of the most widely used software for performing static analyses of mechanical components using the finite element method (FEM) [23,24,25].

The modal analysis provides the primary information related to the verification and validation of the correctness of the frame model setup and its dynamic properties. In addition, modal analysis can indicate eventual shortcomings of the analyzed structure; thus, optimize the structure before production. For the sake of the proposed vehicle as a transportation device, it is indispensable to consider additional dynamic stresses over a relatively wide range of excitation frequencies. For this reason, a modal analysis was performed.

The modal analysis was carried out on a model without defined internal damping. The boundary conditions for the modal analysis were determined in the form of removing all degrees of freedom. The first 6 mode shapes (eigenfrequencies) were examined. In terms of the eigenfrequencies, the maximum response of the structure occurs at the minimum input energy. This may cause excessive wear or even failure of the structure.

From a historical point of view, calculation procedures are known to greatly simplify the nature of the component. Today, the widespread finite element method makes it possible to simplify the procedure for verifying the strength of a component. As it will be seen in Section 4.2, this group of finite elements forms a mesh covering the body. The frame structure is made up of profiles and plates. Therefore, it is convenient to reduce the volume elements to shell elements. The advantage of this step is that the thickness of the shell element can be easily changed. In the case of unsatisfactory results, the parameters of the input material can be rapidly changed. This eliminates the need for manual modification of the model in an external modeling program. The input parameters are the conditions that determine the stress of the model, the material properties, and the input geometry of the structure from the external modeling program. Boundary conditions were defined on the basis of possible situations that arise during the operation of the equipment. Thus, conditions comprised loads from internal components, self-weight, and from the rider along with the seat. The removal of degrees of freedom was made at the points where the vehicle came into contact with the road. Additionally, the material properties were specified as a homogeneous isotropic material. Since it was an aluminum alloy, parameters such as Young’s modulus, Poisson’s ratio, and density were considered in the calculation.

The geometry of the E3-cycle frame was imported to Ansys software from the Catia V5R20 software. Linear tetrahedron elements were used to mesh the model. In terms of numerical calculation, the locations of the stress concentration load induced by the vehicle operation were detected. It was also feasible to determine the values of the bending and torsional deformations that arose. The torsional stiffness as well as the flexural stiffness were determined in the FEM software Ansys. The distinct analyses that were performed on the E3-cycle needed the force applied to the frame. The force, which was employed to analyze the vehicle, depended on the weight of the vehicle (the mass of the E3-cycle concentrated at its center of gravity is 151.5 kg) (Figure 11) and the weight of a model of the rider (rider mass is 130 kg) acting on the vehicle at the time of its operation. Gravitational forces are mainly responsible for bending the frame (also during braking and acceleration in the straight direction [26]), while in the course of cornering, the dominant forces (centrifugal) acting on the vehicle are chiefly related to the torsional stiffness of the vehicle [27].

**Figure 12:**
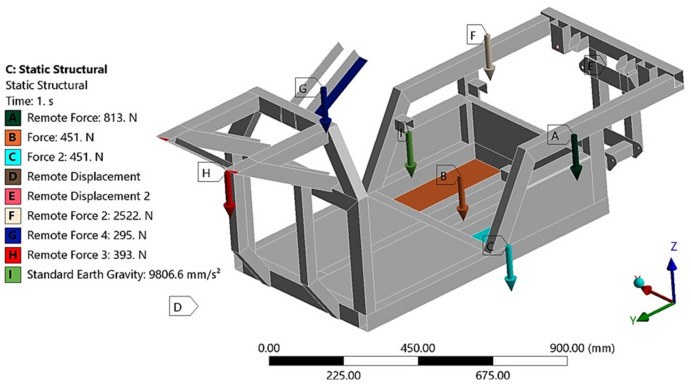
Boundary conditions of the first limit state.

**Figure 13:**
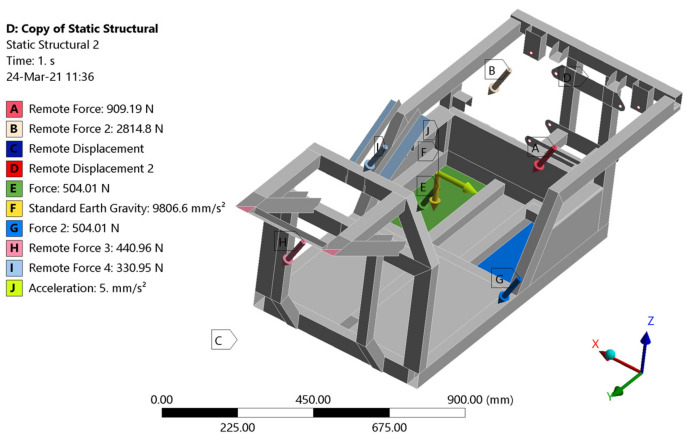
Boundary conditions of the second limit state.

The total mass of the vehicle with the rider was 281.5 kg. Because of safety, a 2∙g acceleration of gravity was considered. Then, the force exerted on the frame is double the weight of the vehicle with rider. The force is subsequently redistributed into the attachments of the frame and vehicle components (explained in Figure 12 and Figure 13). Torsional stiffness expresses how much resistance a given cross-section of the frame structural units produces to the torsional forces acting on it [28,29,30,31]. Flexural analysis helps in determining the ductility of EN AW6063 aluminum alloy and, moreover, how it will resist a break when subjected to loading forces. The torsional stiffness of the frame is one of the crucial factors in its design. Inasmuch as the torsional stiffness of the frame determines its torsion, it significantly influences the suspension design of a vehicle. Flexural stiffness defines the frame efficiency, as the energy lost during acceleration due to chassis bending; this can be discovered by dint of flexural analysis. In vehicle suspension design, the frame is considered to be absolutely rigid. Therefore, it is essential to determine the displacement caused by the torsional forces acting on the chassis.

In terms of determining the stresses by means of static analysis, two limit states were considered. The first state (Figure 12) simulated the calculation of the stresses of the E3-cycle frame at rest and in straight-line motion, respectively. In this case, the frame is not acted upon by centrifugal (transverse) forces arising from the motion of the body on the curved path. The loading forces resulted from the vehicle’s and rider’s own weights. The distribution of stresses in the frame for a freestanding vehicle was investigated. This calculation is only informative due to the fact that the highest stress achieved is essential. The highest stress will certainly be accomplished at the second limit state. The boundary conditions for the first limit state are defined in Figure 12 (mass of the electric motor 41.4 kg, mass of the control electronics 23 kg, mass of the batteries 23 kg, mass of the rider 130 kg, mass of the steering wheel and steering mechanism 15 kg, front axle load 20 kg, mass of the frame 29 kg).

The second limit state (Figure 13) simulated the passage of the analyzed vehicle through a curve with the considered lateral acceleration reaching half the value of the gravitational acceleration *g*. The loading forces were determined with regard to the limiting adhesion of the E3-cycle’s tires to the road. Accordingly, the presented boundary conditions correspond to the actual limit state of the vehicle in question.

### 3.3. Measurement of the Multiaxial Fatigue Life of the Frame Material in a Flexure-Torsion Combination

**Figure 14:**
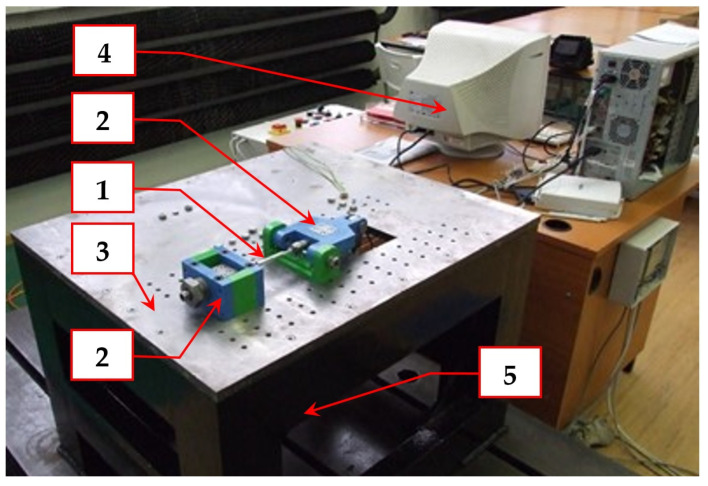
Multiaxial fatigue test system: 1—a specimen, 2—jaws for clamping a specimen, 3—a table (a support) of the test system, 4—a PC controlling and processing system, 5—acting components of the test system (under the table).

The fatigue life measurement of the frame material was carried out on test equipment designed by the authors (Figure 14) and described in detail in papers [12,32,33]. The main components of the test device are depicted in Figure 14.

Testing procedure was conducted in controlled strain amplitude (Manson–Coffin) in a combination of bending and torsion along with a cycle asymmetry coefficient of *R* = −1. The phase shift between loads was *φ* = 0° and 90° under sinusoidal cyclic loading. A symmetric frequency of loading cycles *f* = 30 Hz was employed.

**Figure 15:**
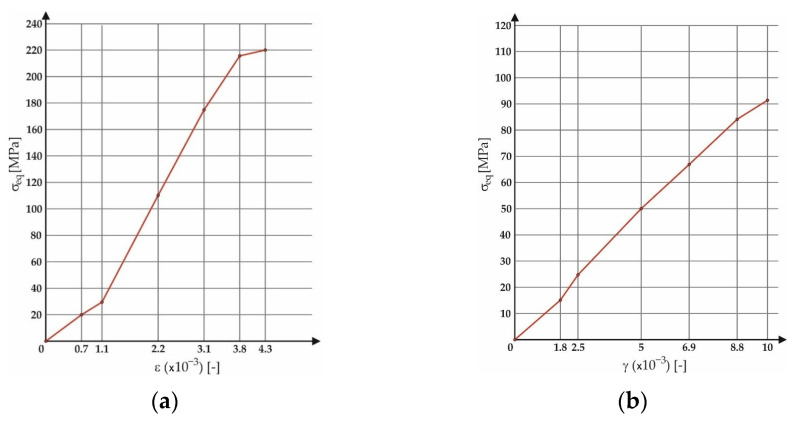
A function of equivalent von Mises stresses of EN AW6063 material tested with the proposed multiaxial test system and subjected to strain: (**a**) In bending; (**b**) In torsion.

The value of strain obtained by the test equipment corresponded to the stress of the test specimens equivalent to the stress of the vehicle frame (and its immediate surroundings) achieved in operation at the second limit state. By means of numerical simulation in Adina (Framingham, MA, USA) [32], calibration curves (Figure 15) were established for the EN AW6063 test material, which give the dependence between the strain amplitude (induced by the test equipment) and the value of the stress attained in the test specimen. This issue was elaborated in detail by the authors in paper [12], and for this reason it is not part of the present paper. Nevertheless, the presented calibration curves (Figure 15) have not been published.

## 4. Results

The section reports the most important analytically, numerically, and experimentally attained results in the field of stability of the designed unconventional vehicle, load capacity, strength, and structural integrity of its frame.

### 4.1. Theoretical Effect of Improving Vehicle Stability

For the sake of a qualitative description of the results, a comparison of the E3-cycle at the same geometrical parameters with the conventional tricycle was made. The comparison was made based on the value of the maximum speed at which the vehicle could move through a corner of a given radius. The stability condition must not be violated; in other words, the wheel to be relieved must still be loaded with a non-zero force. The same safety parameter was also used in paper [34]. For the calculation, the turn radii *R_z_* = 1.5, 5, 10, 15, 20, 25, and 30 m were chosen and substituted to the analytically attained Formulas (6) and (13). Other corresponding input values involved wheelbase *i* = 1.3 m, distance of the vehicle’s center of gravity from the front wheel *i*_1_ = 0.82 m, rear wheel track *b* = 0.725 m, and height of the vehicle’s center of gravity from road surface *h_t_* = 0.3539 m. The distance of the center of gravity of the vehicle from the link of the front and rear wheels (around which the overturning of the vehicle occurs) is a variable value (red dimension *l* in Figure 10), as well as the deflection of the front wheel from the center position (dimension *j* in Figure 10). In addition, the angle *φ* is a variable value (Figure 10). The variation of these parameters depending on the steering wheel rotation angle and the curvature radius of the corner corresponding to that rotation is explained in Table 1.

**Figure 16:**
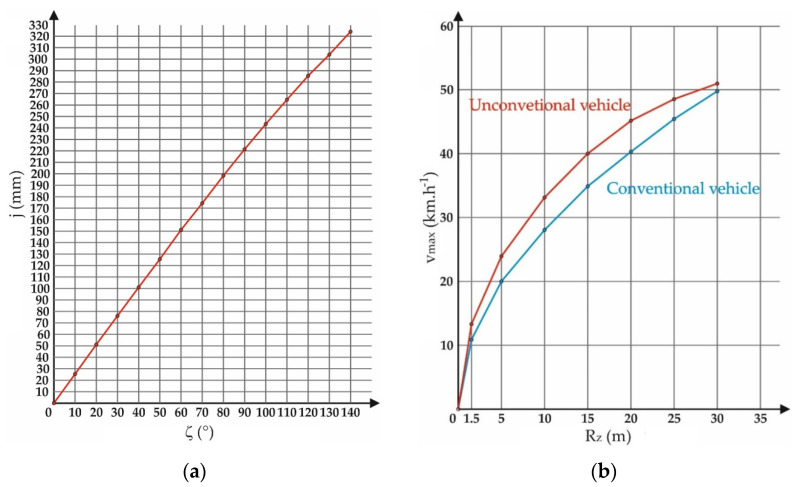
(**a**) Front wheel deflection characteristic *j* as a function of steering wheel angle *ζ*; (**b**) graph of the dependence of the maximum theoretical riding speed *v_max_* on radius of a corner *R_z_*.

Figure 16a depicts the characteristic of front wheel displacement relative to the vehicle’s central axis *j* as a function of the steering wheel angle *ζ*. The deviation of the front wheel is generated by turning the steering wheel via the patented front steered wheel suspension mechanism. The E3-cycle can ride in a minimum curve with the radius of *R_z_* = 1.5 m. The steering wheel rotation of 140° corresponds to that radius. Accordingly, this value of steering wheel rotation angle represents the extreme position of the mechanism. Furthermore, the front wheel is deviated by *j* = 323.9 mm in this position.

Table 2 shows the determined values of the maximum speeds of the two types of vehicles depending on the radius of the corner. The results are clearly processed in the graph in Figure 16b.

The smaller the turning radius, the greater the improvement in stability. This proves that the mechanism primarily fulfills the function for which it was designed.

### 4.2. Results of the Research on the Load-Bearing Capacity of the E3-Cycle Frame

**Figure 17:**
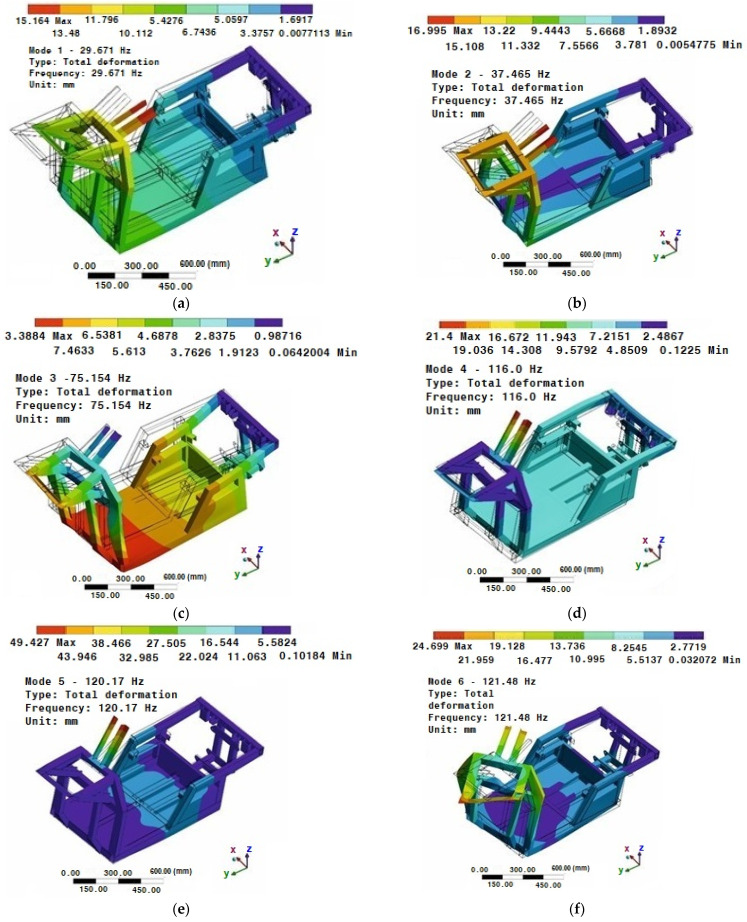
Eigenmodes of the vehicle frame of the E3-cycle: (**a**) first mode; (**b**) second mode; (**c**) third mode; (**d**) fourth mode; (**e**) fifth mode; (**f**) sixth mode.

The eigenfrequency values are given in Table 3 and correspond to the eigenmodes shown in Figure 17. As mentioned earlier, the damping of the system was not defined for the modal analysis. Therefore, the natural frequencies of the oscillations in fact reach slightly lower values.

The results show not only the value of the eigenfrequencies (Table 3), but also the possible deformation of the structure (Figure 17). This deformation is known as the eigenmode of the oscillation. However, it does not take into consideration the excitation function. In order to find out how the structure will react at a certain excitation, it is still necessary to perform the analysis at a certain harmonic excitation function.

**Figure 18:**
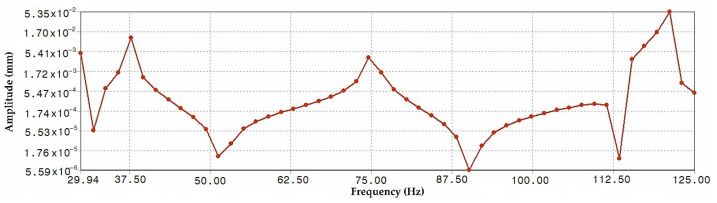
Frequency analysis of the designed E3-cycle frame.

The output of this analysis can be seen in Figure 18. It shows a long frequency analysis of the given design. The curve demonstrates at which frequencies this particular load will deform or vibrate the structure the most. These frequencies are similar to the frequencies of the modal analysis for free oscillation. Nevertheless, they are affected by a given set of loading forces. As a result of the analysis performed, it is known at which frequencies the structure is more vulnerable. Advanced vehicle designers can avoid these frequencies by means of appropriate design modifications. More precisely, frequencies can be shifted to areas where they will not be hazardous to the structure.

**Figure 19:**
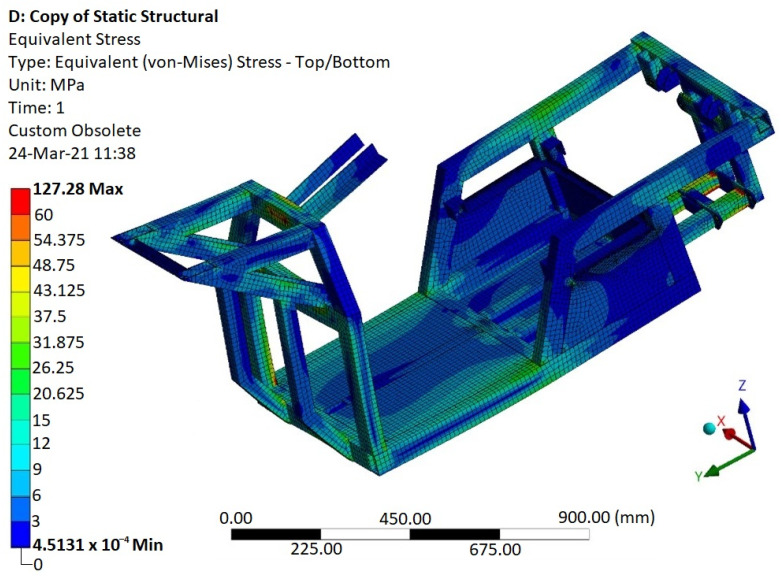
Simulated stresses in the E3-cycle frame in the course of cornering to define the mean stress as an input for fatigue life assessment.

On the basis of the static numerical simulation, it was found that the largest stress of 127 MPa is at the rear of the frame (Figure 19), and it is located at the bottom near the rear axle mount. This value of stress is safe due to the fact that the frame of the E3-cycle is made of the aluminum alloy AW6063 with an ultimate tensile strength *R_m_* = 247 MPa and yield strength *R_e_* = 160 MPa. It needs to be emphasized that this stress will never be exceeded (not even reached due to the consideration of a human mass of 260 kg), for the reason that it occurs at the limiting value of the adhesion of the tire to the road. The average value of the stress in the frame in service will be within the range of 10 to 20 MPa.

**Figure 20:**
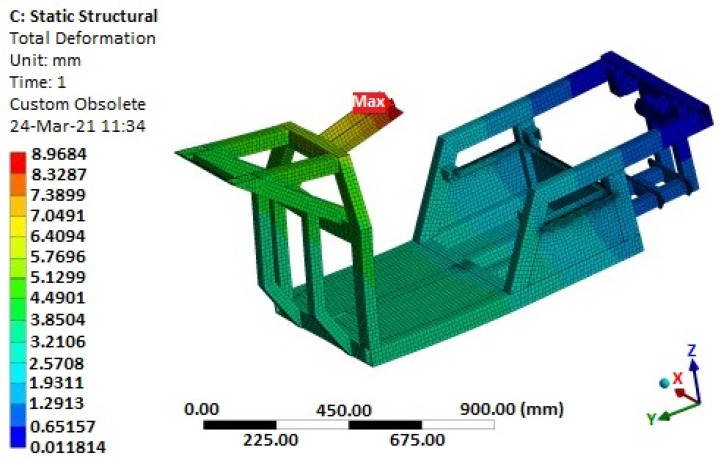
Simulated deformation of the E3-cycle frame at maximum state of stress.

Figure 20 illustrates the analysis of the overall stiffness of the frame. There is no noticeable enormous deflection of the structure, either locally or globally. Thus, a malfunction of some parts of the steering mechanism or deterioration of the vehicle’s riding properties due to the axle-mounting geometry is not foreseen. The designed structure is suitable from the point of view of the determination of the given load forces.

### 4.3. Results of the Research on the Fatigue Life of the Frame Material of the E3-Cycle

The measured and subsequently calculated average values of the number of cycles to fatigue fracture of the experimental specimens (the testing conditions, specimen geometry, and description of the test equipment were reported in [10,16,32]) are shown in Table 4 and Table 5.

The calibration of the test equipment was checked by means of comparing the experimentally obtained results with the results provided by the Fatigue Calculator program (Altair, Troy, MI, USA) [35]. Generally, Fatigue Calculator is a freely available program from the eFatigue website. It was created at the University of Illinois. Fatigue Calculator performs fatigue life estimation rapidly and easily. The comparison of the experimental results of low-cycle fatigue was carried out with the Brown–Miller, Fatemi–Socie and Liu SWT damage models [36,37,38,39]. Finite element method simulation using the Adina software was used to get the stress values in the test specimens as a function of strain amplitude. This was published by the authors in paper [12]. With respect to this paper, it is indispensable to set the strain in the interval *ε_xx_* = 0.5∙10^−3^ to 1.1∙10^−3^ (according to the curves shown in Figure 15b) on the test equipment in order to achieve the intermediate values of the stress in the frame (10 to 20 MPa) for the flexural load. In the case of torsion, strain values in the range *γ_xy_* = 1∙10^−3^ to 2.5∙10^−3^ are essential.

**Figure 21:**
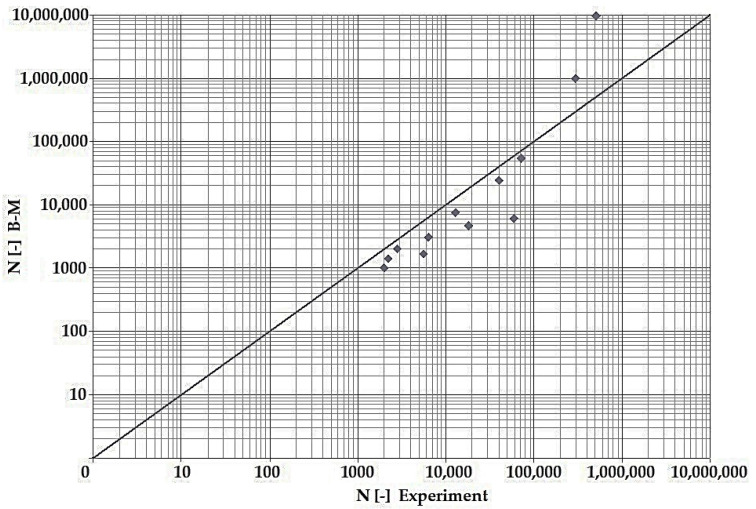
Dependence of theoretical and experimentally obtained number of cycles, Brown–Miller vs. experiment, phase shift 0°.

**Figure 22:**
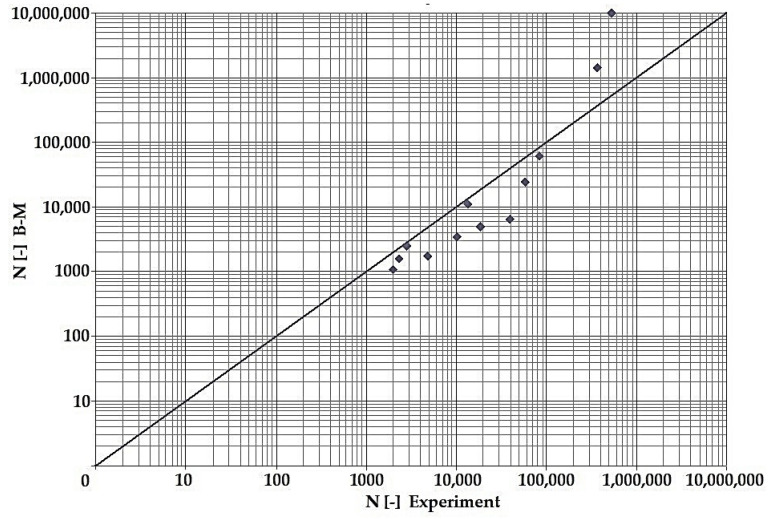
Dependence of theoretical and experimentally obtained number of cycles, Brown–Miller vs. experiment, phase shift 90°.

**Figure 23:**
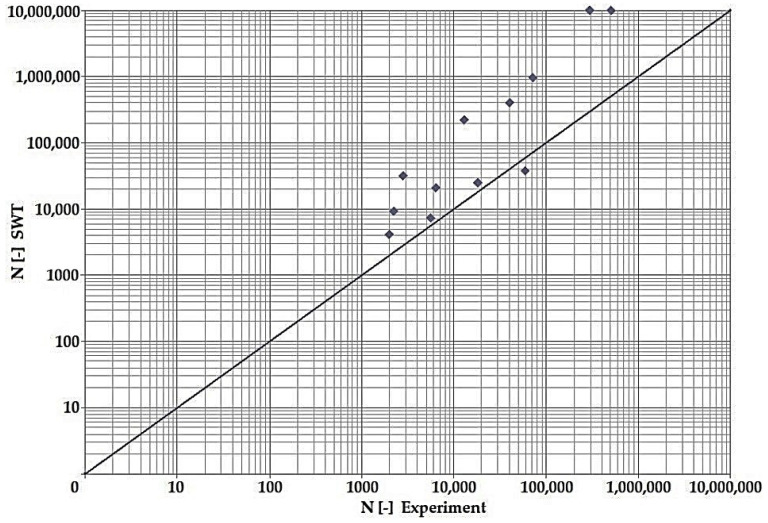
Dependence of theoretical and experimentally obtained number of cycles, SWT vs. experiment, phase shift 0°.

**Figure 24:**
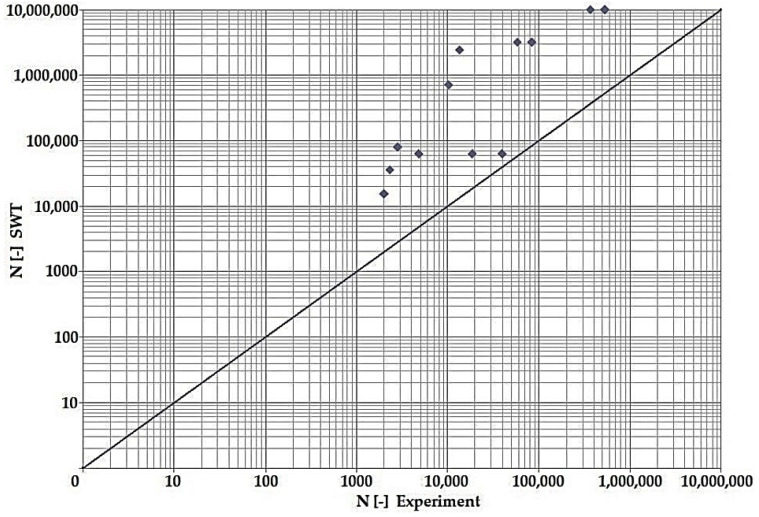
Dependence of theoretical and experimentally obtained number of cycles, SWT vs. experiment, phase shift 90°.

**Figure 25:**
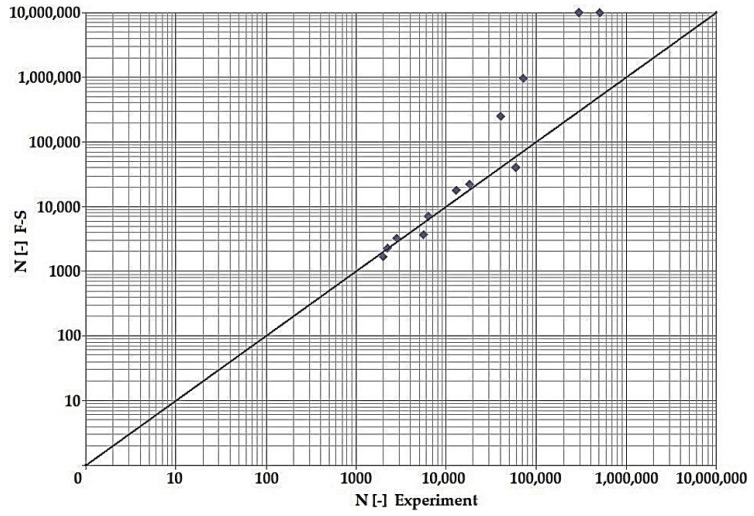
Dependence of theoretical and experimentally obtained number of cycles, F–S vs. experiment, phase shift 0°.

**Figure 26:**
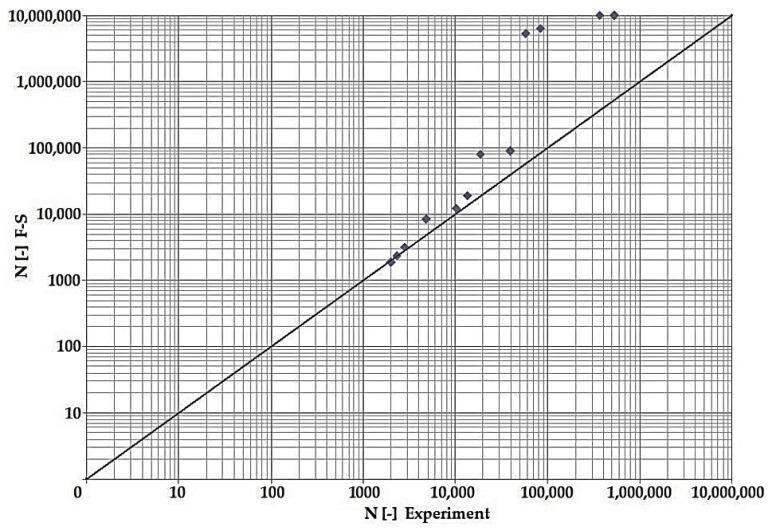
Dependence of theoretical and experimentally obtained number of cycles, F–S vs. experiment, phase shift 90°.

**Figure 27:**
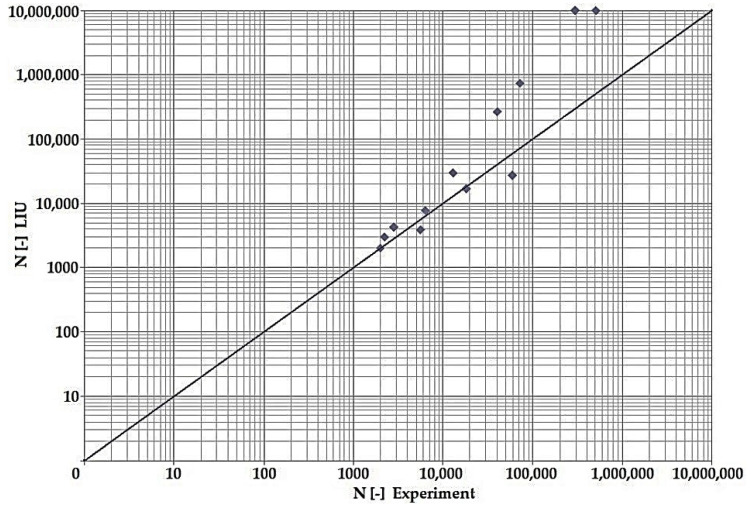
Dependence of theoretical and experimentally obtained number of cycles, Liu vs. experiment, phase shift 0°.

**Figure 28:**
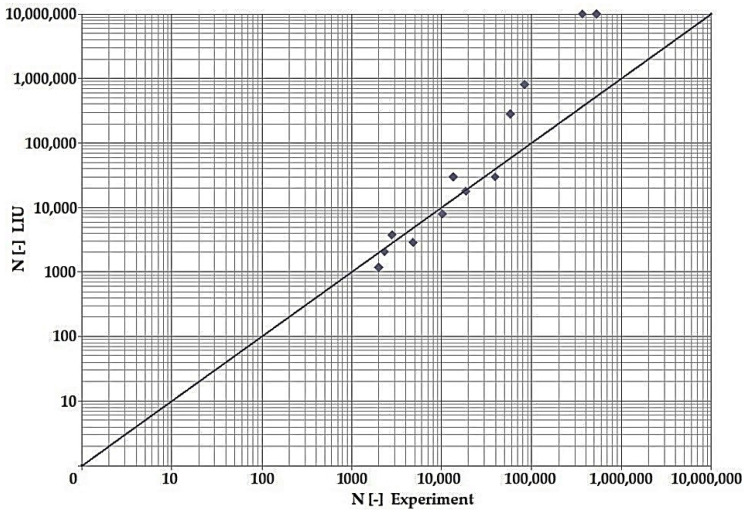
Dependence of theoretical and experimentally obtained number of cycles, Liu vs. experiment, phase shift 90°.

In terms of the dependencies (Figure 21, Figure 22, Figure 23, Figure 24, Figure 25, Figure 26, Figure 27 and Figure 28), a quite satisfactory match in all criteria with the experiment can be seen in the low-cycle fatigue region (up to 105). Moreover, experimental measurement was also performed in the high-cycle fatigue region (results of number of cycles to fracture more than 105). The variance of values is already considerable in this case. The reason for the variance is that the selected criteria (F-S, B-M, SWT, Liu) are employed only for the evaluation of low-cycle fatigue.

Damage models such as Goodman’s, Findley’s, Sines’, Dang Van’s, and the MCE model are used in high-cycle fatigue [40]. The lifetime results for the Fatemi–Socie (Figure 25 and Figure 26), Brown–Miller (Figure 21 and Figure 22), and Liu (Figure 27 and Figure 28) damage models demonstrate a considerable match of the courses over the entire range of the monitored number of cycles for low-cycle fatigue. In terms of the SWT model (Figure 23 and Figure 24), a significant but uniform increase in fatigue life was observed in contrast to the other models in the region of low-cycle load.

The phase shift of loads (90°, flexure-torsion) will cause a gain in fatigue life of approximately 10% compared to testing without phase shift (0°). Therefore, in the case of assessing the fatigue life of the frame material, the minimum values of the number of cycles resulting from the most unfavorable states are taken into account.

By dint of creating graphs representing the dependency between the number of cycles to the fatigue fracture of the criteria used and the measured number of cycles from the experiment at the same load levels, we can simply and rapidly follow the relative differences between cycles. This is ensured by means of creating a straight line at a 45° angle in the graph. If the hypotheses along with the experiment provide the same results, the intersections of the lines drawn from the limiting number of cycles to the fracture at the same load level meet just on the line mentioned above. The greater the difference in the number of cycles to fracture between the hypothesis and the experiment, the greater will be the distance of the intersections from the straight line.

## 5. Discussion

The authors’ efforts to construct a safe and functional unconventional vehicle show what an interdisciplinary problem this really is. The results of the stability of the unconventional vehicle show (Figure 16b) that the designed mechanism is theoretically functional. In terms of vehicles of the same parameters (in conventional and unconventional designs), the proposed mechanism gains vehicle cornering speed at the boundary condition *R_zmin_* = 1.5 m by up to 19%. Research into vehicle stability is also investigated and presented in [41]. The authors carried out an extensive analysis in order to compare the advantages of other authors’ mechanisms [42,43,44,45,46,47,48]. The paper [42] discussed the mathematical modeling of a tricycle, namely the E3-cycle, with the possibility of rotating the front wheel around the vertical axis of the vehicle. Accordingly, the authors attributed to this motion the expected improvement in riding characteristics based on a combination of the advantages of two-wheeled and four-wheeled vehicles. They attained this by means of a hinge joint in the center of the vehicle. The expected benefits of the design are identical to the E3-cycle system. However, the E3-cycle system works on a completely diverse mechanical principle and the front wheel has different movement possibilities at the same time.

The authors in the paper [43] compared, among other things, the cornering stability of a three-wheeled vehicle with one wheel in front (1F2R). It was established that the stability of a three-wheeled vehicle can be increased by introducing a tilting mechanism. They achieved a theoretical gain in the critical speed value from 17 km∙h^−1^ (without the tilting mechanism) to 27 km∙h^−1^ (at a corner radius of 50 m). In terms of comparison, the E3-cycle at this speed value raised the cornering speed from 20 km∙h^−1^ to 24.5 km∙h^−1^, at a radius of the corner of 5 m. An exact qualitative comparison of the mechanisms is not possible to make, inasmuch as each of them was implemented in a vehicle with a different geometry (wheelbase, track width, center of gravity height, and so forth).

Several authors have approached the issue of stability from other perspectives. Nonetheless, all the solutions traced have a common base, namely a significant effort to trigger the tilting actuator [44], or other complicated solutions presented, for example, in papers [45,46]. Analysis was also performed within the patent office. The issue of increasing the stability of tricycles has been addressed, for instance, by the Slovak utility model [47], where the essential feature of the described solution is the fitting of a front wheel fork into a rotatably mounted directional member and joint. Nevertheless, the disadvantage of this solution is that it is intended for three-wheeled motorcycles with motorcycle-type steering, specifically forked handlebars, which cannot be used for safe steering of the vehicle due to the relatively large forces generated in the course of cornering and passing through a curve at higher speeds. It is necessary to overcome the relatively large forces on the handlebars when returning to a straight line. In terms of the E3-cycle, this problem is eliminated by the structural modifications introduced and the specially designed shape of the tire of the front wheel. The E3-cycle will be more economically advantageous because of its simple mechanical system. Some works have addressed the stability of tricycles in terms of the problem of vehicle rollover due to striking an obstacle [48]. However, this is not the subject of the present paper. Currently, there is a lack of other papers involving mechanisms increasing the stability of tricycles based on the front steered wheel deflection during cornering. This ensures the originality of the paper in terms of a design.

Modal (Figure 17), frequency (Figure 18), static (Figure 19), and stiffness analysis (Figure 19) were performed to verify the vehicle frame. The frame was specifically designed to attach the patented unconventional steering mechanism of the front wheel of the vehicle. In the research papers searched [22,26,27,28,49,50,51] (and there are certainly incomparably more of them), the same analyzes were used for the authors’ purposes with the intention of analyzing their own designs. This was carried out without the need to develop the known computational procedures of the mentioned analyses or computer programs. Nonetheless, the above-mentioned research papers were an essential part of all the research work, inasmuch as they appropriately complemented and clarified the issues addressed, and thus made the designs “viable”.

The issue of the fatigue life research of the frame material is also a crucial part of the paper and has its theoretical contribution in the fields of mechanics, technology, and materials. The methodology of test specimen fabrication and the development of test equipment were published by the authors in papers [10,12,16]. The same methodology and test equipment were employed for the current research. The calibration curve (Figure 15) was created for the test material EN AW6063, indicating the dependence of the attained stress (bending, torsion) on the set strain amplitude (*ε*, *γ*). The experimentally obtained results (Figure 21, Figure 22, Figure 23, Figure 24, Figure 25, Figure 26, Figure 27 and Figure 28) are used directly in the design of the structural unit being solved. The authors attempted to get as close as possible to the real loading of the frame in service by the experiment carried out on the test specimens.

The analysis of the current state of this issue leads to the conclusion that there is considerable room in the literature for the improvement of the fatigue life of three-wheeled vehicle frames. There is a lack of studies dealing with such an application. The vast majority of the papers attend to research on the fatigue life of rolling stock frames or the materials themselves, or the papers are exclusively focused on improving the effectiveness of the criteria themselves. For example, in papers [52,53] the authors scrutinize the fatigue life of the bogie frame of the metro vehicle using a laboratory test bench. In particular, motor traction vibrations are the subject of interest. However, there are no devices in the E3-cycle that induce specific excitation vibrations that would cause the resonance of the system, and thus damage to the frame. In paper [54], the fatigue life of a rail vehicle frame is scrutinized directly by means of field measurements. Petrone and Meneghetti [55] examined the fatigue life of a moped frame also by means of field measurements using strain gauges compared to the FEM simulation used to solve the E3-cycle frame. However, all contact measurement methods affect the measured object, and thus change its properties and results to a greater or lesser extent. Gu et al. [56] used a similar methodology in comparison to the E3-cycle research. The object of interest was a mining vehicle, where the results of the simulations were verified through experiments on a real vehicle. This is not yet possible for the E3-cycle because it is in the design and optimization phase. The first real prototype made is functional and safe in terms of load-carrying capacity. Fatigue life has not yet been realistically investigated due to the small number of kilometers ridden (approximately 50 km).

## 6. Conclusions

During the process of creating a new type of three-wheeled means of transport, the following things were proposed:

- two further design modifications of the original steering mechanism;
- a special design of the front steered wheel tire;
- a stress-strain calibration curve of the test equipment for the EN AW6063 material of the vehicle frame;
- a linear mathematical model of the addressed vehicle in the course of cornering;
- a methodology for testing the suitability of the vehicle frame for the unconventional steering mechanism.

During the process of creating the new type of three-wheeled means of transport, it was found that:

- the designed patented front wheel steering mechanism increased the passing speed by 19% in comparison with a conventional vehicle at the minimum possible radius of a corner;
- riding on the proposed vehicle has a positive effect on ride comfort and safety;
- the designed vehicle fulfills safety conditions in terms of frame integrity and load capacity;
- the vehicle frame is designed with respect to the fatigue life of the material; for this reason, it is serviceable;
- the authors enriched the stability, development, and durability of the frames of unconventional three-wheeled vehicles.

## Figures and Tables

**Table 1:** Variable parameters of the E3-cycle depending on steering wheel angle.

Radius of a Corner	Rotation Angle of Steering Wheel from Its Center Position	Deflection of the Front Wheel from the Center Position	Distance of the Center of Gravity of the Vehicle from the Link of Front and Rear Wheels	Angle of Deflection of the Link of the Front and Rear Wheels of the E3-Cycle from the Vertical Axis
*R_z_* (m)	*ζ* (°)	*j* (m)	*l* (m)	*φ* (°)
1.5	140	0.3239	0.3481	1.7
5	120	0.2858	0.3336	3.37
10	100	0.2439	0.3174	5.21
15	80	0.1987	0.2997	7.18
20	60	0.1518	0.2810	9.206
25	40	0.1016	0.2610	11.34
30	20	0.0511	0.2407	13.47

**Table 2:** Comparison of the theoretically obtained maximum vehicle speeds in the course of cornering.

Radius of a Corner	Maximum Speed of a Conventional Vehicle	Maximum Speed of the E3-Cycle	Percentage Expression of Speed Increase in Unconventional Vehicle versus Conventional Vehicle
*R_z_* (m)	(km∙h^−1^)	(km∙h^−1^)	(%)
1.5	11.1	13.69	19
5	20.3	24.5	17
10	28.66	33.83	15
15	35.1	40.34	13
20	40.53	45.21	10
25	45.3	48.8	7
30	49.6	51.62	4

**Table 3:** Eigenfrequencies of oscillation of the E3-cycle frame, undamped oscillation.

Eigenfrequency		
Eigenmode	Designation	Value (Hz)
1st	*f*_1_	29.671
2nd	*f*_2_	37.465
3rd	*f*_3_	75.154
4th	*f*_4_	116.00
5th	*f*_5_	120.17
6th	*f*_6_	121.48

**Table 4:** Determined average number of cycles to fracture of the frame material at phase shift 0°.

		*γ* = 0 × 10^−3^	*γ* = 1.8 × 10^−3^	*γ* = 2.5 × 10^−3^	*γ* = 5 × 10^−3^	*γ* = 6.9 × 10^−3^	*γ* = 8.8 × 10^−3^	*γ* = 10 × 10^−3^
**Experiment**	*ε* = 0 × 10^−3^	-	4.2 × 10^6^	6.4 × 10^5^	5.4 × 10^4^	5.4 × 10^3^	1.3 × 10^3^	6.9 × 10^2^
**B-M**		-	1 × 10^7^	1 × 10^7^	2.1 × 10^4^	3.1 × 10^3^	1.3 × 10^3^	8.7 × 10^2^
**SWT**		-	1 × 10^7^	1 × 10^7^	3.3 × 10^6^	1.5 × 10^5^	3.6 × 10^4^	1.8 × 10^4^
**F-S**		-	1 × 10^7^	1 × 10^7^	3.2 × 10^4^	4.1 × 10^3^	1.6 × 10^3^	1.1 × 10^3^
**LIU**		-	1 × 10^7^	1 × 10^7^	5.7 × 10^4^	5 × 10^3^	2 × 10^3^	1.3 × 10^3^
**Experiment**	*ε* = 0.7 × 10^−3^	2.9 × 10^6^	1.7 × 10^6^	5.1 × 10^5^	4 × 10^4^	3.5 × 10^3^	1.1 × 10^3^	6.4 × 10^2^
**B-M**		1 × 10^7^	1 × 10^7^	4.9 × 10^6^	1.1 × 10^4^	2.4 × 10^3^	1.1 × 10^3^	7.8 × 10^2^
**SWT**		1 × 10^7^	1 × 10^7^	1 × 10^7^	5.9 × 10^5^	5.4 × 10^4^	1.8 × 10^4^	1 × 10^4^
**F-S**		1 × 10^7^	1 × 10^7^	1 × 10^7^	2.3 × 10^4^	3.6 × 10^3^	1.5 × 10^3^	1 × 10^3^
**LIU**		1 × 10^7^	1 × 10^7^	1 × 10^7^	4.3 × 10^4^	4.7 × 10^3^	1.9 × 10^3^	1.3 × 10^3^
**Experiment**	*ε* = 1.1 × 10^−3^	6.3 × 10^5^	5.1 × 10^5^	2.9 × 10^5^	1.3 × 10^4^	3.5 × 10^3^	1.1 × 10^3^	6.4 × 10^2^
**B-M**		1 × 10^7^	9.6 × 10^6^	1 × 10^6^	7.5 × 10^3^	2.4 × 10^3^	1.1 × 10^3^	7.8 × 10^2^
**SWT**		1 × 10^7^	1 × 10^7^	1 × 10^7^	2.2 × 10^5^	5.4 × 10^4^	1.8 × 10^4^	1 × 10^4^
**F-S**		1 × 10^7^	1 × 10^7^	1 × 10^7^	1.8 × 10^4^	3.6 × 10^3^	1.5 × 10^3^	1 × 10^3^
**LIU**		1 × 10^7^	1 × 10^7^	1 × 10^7^	3 × 10^4^	4.7 × 10^3^	1.9 × 10^3^	1.3 × 10^3^
**Experiment**	*ε* = 2.2 × 10^−3^	1.5 × 10^5^	7.2 × 10^4^	4 × 10^4^	6.3 × 10^3^	2.2 × 10^3^	7 × 10^2^	5.4 × 10^2^
**B-M**		2 × 10^5^	5.4 × 10^4^	2.4 × 10^4^	3.1 × 10^3^	1.4 × 10^3^	8 × 10^2^	6.2 × 10^2^
**SWT**		3.1 × 10^6^	9.7 × 10^5^	4.1 × 10^5^	2.1 × 10^4^	9.4 × 10^3^	4.9 × 10^3^	3.4 × 10^3^
**F-S**		7.5 × 10^6^	9.8 × 10^5^	2.5 × 10^5^	7.2 × 10^3^	2.3 × 10^3^	1.2 × 10^3^	8.8 × 10^2^
**LIU**		2.8 × 10^6^	7.4 × 10^5^	2.7 × 10^5^	7.8 × 10^3^	3 × 10^3^	1.5 × 10^3^	1.1 × 10^3^
**Experiment**	*ε* = 3.1 × 10^−3^	7.3 × 10^4^	5.9 × 10^4^	1.8 × 10^4^	5.6 × 10^3^	2 × 10^3^	6.4 × 10^2^	5.1 × 10^2^
**B-M**		9.4 × 10^3^	6.2 × 10^3^	4.7 × 10^3^	1.7 × 10^3^	1 × 10^3^	6.5 × 10^2^	5.2 × 10^2^
**SWT**		6.4 × 10^4^	3.8 × 10^4^	2.5 × 10^4^	7.3 × 10^3^	4.2 × 10^3^	2.7 × 10^3^	2.1 × 10^3^
**F-S**		1 × 10^5^	4 × 10^4^	2.2 × 10^4^	3.7 × 10^3^	1.7 × 10^3^	1 × 10^3^	7.7 × 10^2^
**LIU**		5.2 × 10^4^	2.7 × 10^4^	1.7 × 10^4^	3.9 × 10^3^	2 × 10^3^	1.2 × 10^3^	9.6 × 10^2^
**Experiment**	*ε* = 3.8 × 10^−3^	2.3 × 10^4^	9.9 × 10^3^	6.5 × 10^3^	3.4 × 10^3^	1.5 × 10^3^	6.1 × 10^2^	4.8 × 10^2^
**B-M**		3.2 × 10^3^	2.6 × 10^3^	2.2 × 10^3^	1.2 × 10^3^	7.9 × 10^2^	5.6 × 10^2^	4.6 × 10^2^
**SWT**		9.8 × 10^3^	7.3 × 10^3^	6.4 × 10^3^	3.9 × 10^3^	2.6 × 10^3^	1.8 × 10^3^	1.5 × 10^3^
**F-S**		1.4 × 10^4^	9.3 × 10^3^	6.8 × 10^3^	2.4 × 10^3^	1.3 × 10^3^	8.6 × 10^2^	6.8 × 10^2^
**LIU**		7.5 × 10^3^	5.5 × 10^3^	4.6 × 10^3^	2.5 × 10^3^	1.6 × 10^3^	1 × 10^3^	8.3 × 10^2^
**Experiment**	*ε* = 4.3 × 10^−3^	1.1 × 10^4^	7.1 × 10^3^	3.6 × 10^3^	2.1 × 10^3^	9.3 × 10^2^	4.7 × 10^2^	4.5 × 10^2^
**B-M**		1.9 × 10^3^	1.7 × 10^3^	1.5 × 10^3^	9.5 × 10^2^	6.8 × 10^2^	5 × 10^2^	4.2 × 10^2^
**SWT**		4.7 × 10^3^	4.3 × 10^3^	3.9 × 10^3^	2.7 × 10^3^	1.9 × 10^3^	1.4 × 10^3^	1.2 × 10^3^
**F-S**		6 × 10^4^	4.7 × 10^3^	3.8 × 10^3^	1.8 × 10^3^	1.1 × 10^3^	7.8 × 10^2^	6.3 × 10^2^
**LIU**		3.7 × 10^3^	3.3 × 10^3^	3 × 10^3^	1.9 × 10^3^	1.3 × 10^3^	9.1 × 10^2^	7.5 × 10^2^

**Table 5:** Determined average number of cycles to fracture of the frame material at phase shift 90°.

		*γ* = 0 × 10^−3^	*γ* = 1.8 × 10^−3^	*γ* = 2.5 × 10^−3^	*γ* = 5 × 10^−3^	*γ* = 6.9 × 10^−3^	*γ* = 8.8 × 10^−3^	*γ* = 10 × 10^−3^
**Experiment**	*ε* = 0 × 10^−3^	-	4.2 × 10^6^	6.4 × 10^5^	5.4 × 10^4^	5.4 × 10^3^	1.3 × 10^3^	6.9 × 10^2^
**B-M**		-	1 × 10^7^	1 × 10^7^	2.1 × 10^4^	3.1 × 10^3^	1.3 × 10^3^	8.7 × 10^2^
**SWT**		-	1 × 10^7^	1 × 10^7^	3.3 × 10^6^	1.5 × 10^5^	3.6 × 10^4^	1.8 × 10^4^
**F-S**		-	1 × 10^7^	1 × 10^7^	3.2 × 10^4^	4.1 × 10^3^	1.6 × 10^3^	1.1 × 10^3^
**LIU**		-	1 × 10^7^	1 × 10^7^	5.7 × 10^4^	5 × 10^3^	2 × 10^3^	1.3 × 10^3^
**Experiment**	*ε* = 0.7 × 10^−3^	2.9 × 10^6^	2 × 10^6^	5.4 × 10^5^	4.7 × 10^4^	3.7 × 10^3^	1.3 × 10^3^	6.8 × 10^2^
**B-M**		1 × 10^7^	1 × 10^7^	9.7 × 10^6^	1.5 × 10^4^	2.8 × 10^3^	1.2 × 10^3^	8.4 × 10^2^
**SWT**		1 × 10^7^	1 × 10^7^	1 × 10^7^	2.9 × 10^6^	1 × 10^5^	2.1 × 10^4^	1.5 × 10^4^
**F-S**		1 × 10^7^	1 × 10^7^	1 × 10^7^	2.4 × 10^4^	3.6 × 10^3^	1.5 × 10^3^	1 × 10^3^
**LIU**		1 × 10^7^	1 × 10^7^	1 × 10^7^	4.4 × 10^4^	4.5 × 10^3^	1.9 × 10^3^	1.3 × 10^3^
**Experiment**	*ε* = 1.1 × 10^−3^	6.3 × 10^5^	5.3 × 10^5^	3.7 × 10^5^	1.4 × 10^4^	2.8 × 10^3^	1.2 × 10^3^	6.8 × 10^2^
**B-M**		1 × 10^7^	1 × 10^7^	1.4 × 10^6^	1.1 × 10^4^	2.5 × 10^3^	1.1 × 10^3^	8.1 × 10^2^
**SWT**		1 × 10^7^	1 × 10^7^	1 × 10^7^	2.4 × 10^5^	8.1 × 10^4^	1.9 × 10^4^	1 × 10^4^
**F-S**		1 × 10^7^	1 × 10^7^	1 × 10^7^	1.9 × 10^4^	3.1 × 10^3^	1.3 × 10^3^	9 × 10^2^
**LIU**		1 × 10^7^	1 × 10^7^	1 × 10^7^	3 × 10^4^	3.8 × 10^3^	1.7 × 10^3^	1.2 × 10^3^
**Experiment**	*ε* = 2.2 × 10^−3^	1.5 × 10^5^	8.3 × 10^4^	5.8 × 10^4^	1.1 × 10^4^	2.3 × 10^3^	7.5 × 10^2^	5.8 × 10^2^
**B-M**		2 × 10^5^	6.1 × 10^4^	2.4 × 10^4^	3.5 × 10^3^	1.6 × 10^3^	9 × 10^2^	6.8 × 10^2^
**SWT**		3.2 × 10^6^	3.2 × 10^6^	3.8 × 10^6^	7.2 × 10^5^	3.5 × 10^4^	1 × 10^4^	6.3 × 10^3^
**F-S**		7.5 × 10^6^	6.3 × 10^6^	5.4 × 10^6^	1.2 × 10^4^	2.4 × 10^3^	1 × 10^3^	7.2 × 10^2^
**LIU**		2.9 × 10^6^	8 × 10^5^	2.8 × 10^5^	8 × 10^3^	2.1 × 10^3^	1 × 10^3^	7.7 × 10^2^
**Experiment**	*ε* = 3.1 × 10^−3^	7.3 × 10^4^	3.9 × 10^4^	1.9 × 10^4^	4.8 × 10^3^	2 × 10^3^	6.9 × 10^2^	5.1 × 10^2^
**B-M**		9.4 × 10^3^	6.5 × 10^4^	4.9 × 10^3^	1.7 × 10^3^	1 × 10^3^	7.2 × 10^2^	5.7 × 10^2^
**SWT**		6.4 × 10^5^	6.4 × 10^5^	6.4 × 10^4^	6.3 × 10^4^	1.6 × 10^4^	7 × 10^3^	4 × 10^3^
**F-S**		1 × 10^5^	9 × 10^4^	8.1 × 10^4^	8.5 × 10^3^	1.9 × 10^3^	8.7 × 10^2^	6.4 × 10^2^
**LIU**		5.2 × 10^4^	2.9 × 10^4^	1.8 × 10^4^	2.9 × 10^3^	1.2 × 10^3^	7 × 10^2^	5.7 × 10^2^
**Experiment**	*ε* = 3.8 × 10^−3^	2.3 × 10^4^	1.2 × 10^4^	7.3 × 10^3^	3.7 × 10^3^	1.5 × 10^3^	6.4 × 10^2^	4.9 × 10^2^
**B-M**		3.2 × 10^3^	2.7 × 10^3^	2.3 × 10^3^	1.2 × 10^3^	8 × 10^2^	5.9 × 10^2^	4.9 × 10^2^
**SWT**		2.8 × 10^3^	9.9 × 10^3^	9.8 × 10^3^	9,7 × 10^3^	7.2 × 10^3^	4 × 10^3^	2.7 × 10^3^
**F-S**		1.4 × 10^4^	1.3 × 10^4^	1.3 × 10^4^	6.8 × 10^3^	1.7 × 10^3^	8.3 × 10^2^	6.1 × 10^2^
**LIU**		7.5 × 10^3^	5.7 × 10^3^	4.5 × 10^3^	1.5 × 10^3^	7.7 × 10^2^	5.3 × 10^3^	4.5 × 10^2^
**Experiment**	*ε* = 4.3 × 10^−3^	1.1 × 10^4^	7.7 × 10^3^	5.2 × 10^3^	3 × 10^3^	9.5 × 10^2^	4.8 × 10^2^	4.6 × 10^2^
**B-M**		1.9 × 10^3^	1.7 × 10^3^	1.6 × 10^3^	9.6 × 10^2^	6.8 × 10^2^	5.1 × 10^2^	4.2 × 10^2^
**SWT**		4.6 × 10^3^	4.6 × 10^3^	4.6 × 10^3^	4.6 × 10^3^	3.9 × 10^3^	3.1 × 10^3^	2.3 × 10^3^
**F-S**		6 × 10^3^	5.5 × 10^3^	5.2 × 10^3^	4.4 × 10^3^	1.6 × 10^3^	8.2 × 10^2^	6 × 10^2^
**LIU**		3.7 × 10^3^	2.8 × 10^3^	2.4 × 10^3^	1.1 × 10^3^	6.4 × 10^2^	4.7 × 10^2^	4 × 10^2^

## Data Availability

Not applicable.

## Nomenclature

1F2R	Three-wheeled vehicle with one steered wheel in front and two wheels in the rear
3D	Three-dimensional model
B–M	Fatigue criterion according to Brown and Miller
CAD	Computer-aided design
EU	European Union
FEA	Finite element analysis
FEM	Finite element method
F-S	Fatigue criterion Fatemi–Socie
*F_o_*	Centrifugal force acting on the vehicle during cornering
*G*	Weight of the vehicle
LIU	Fatigue criterion according to Liu
MPa	MegaPascal
MBS	Multibody simulation
*N*	Number of cycles to fatigue fracture of the experimental specimens
*R_e_*	Yield strength of the frame material
*R_m_*	Tensile strength of the frame material
*R_z_*	Radius of a corner
SUV	Sport utility vehicle
SWT	Smith–Watson–Topper fatigue criterion
*T*	Centre of gravity of the vehicle
*α*	Constant angle given by the design of a conventional three-wheeled vehicle
*β*	Variable angle dependent upon the value of the wheel deflection *j*
*γ*	Total shear strain of the test specimen
*δ*	Variable angle dependent upon the value of the wheel deflection *j*
*ε*	Total normal strain of the test specimen
*ζ*	Steering wheel angle
*λ*	Constant angle given by the design of a conventional three-wheeled vehicle (equivalent to angle α)
*μ*	Variable angle dependent upon the value of the wheel deflection *j* (equivalent to the angle *φ*)
*σ_eq_*	Equivalent stress of von Mises
*φ*	Variable angle dependent upon the value of the wheel deflection *j*
*a*	Acceleration of the vehicle
*b*	Track width of the vehicle
*g*	Gravitational acceleration
*h_t_*	Height of the vehicle’s center of gravity above the ground
*i*	Wheelbase of the vehicle
*i*_1_	Distance of the vehicle’s center of gravity from the centerline of the front wheel
*i*_2_	Distance of the vehicle’s center of gravity from the centerline of the rear wheel
*j*	Variable distance of the deflection of the front wheel of the vehicle from the longitudinal axis of symmetry
*l*	Geometric variable dependent upon the parameter *w*
*m*	Mass of the vehicle
*v*	Instantaneous speed of the vehicle
*v_max_*	Maximum (safe) speed of the vehicle moving on the bend
*x*, *y*, *z*	Axes of the coordinate system
*w*	Variable dependent upon the value of the wheel deflection *j*
